# 

**Published:** 1894-06

**Authors:** 


Mr. W. B. Saunders, Medical Publisher, Philadelphia, is pleased to announce, as in active preparation, his New Aid Series of Manuals for Students and Practitioners. As publisher of the Standard Series of Question Compends, together with an intimate relation with leading members of the medical profession, Mr. Saunders has been enabled to study, progressively, the essential *desideratum* in practical self-helps for students and physicians.

This study has manifested that, while the published Question Compends earn the highest appreciation of students, whom they serve in reviewing their studies preparatory to examination, there is special need of thoroughly reliable hand-books on the leading branches of medicine and surgery, each subject being compactly and authoritatively written, and exhaustive in detail, without the introduction of cases and foreign subject-matter which so largely expand ordinary text-books.

The Saunders' Aid Series will not merely be condensations from present literature, but will be ably written by well-known authors and practitioners, most of them being teachers in representative American colleges. This new series, therefore, will form an admirable collection of advanced lectures, which will be invaluable aids to students in reading and in comprehending the contents of "recommended" works.

Each manual, comprising about 250 pages (5½ x 8 inches), will be distinguished further by the beauty of the new type; by the quality of the paper and printing; by the copious use of illustrations; by the attractive binding in cloth; and by the extremely low price, which will uniformly be $1.25 per volume.

---

The Transactions of the Pan-American Medical Congress.—The proceedings of the first Pan-American Medical Congress were compiled by the Secretary-General, Dr. Charles A. L. Reed, and transmitted to the Department of State in November, 1893. By recent joint resolution of the Senate and House of Representatives, the manuscript was transmitted to Congress and a concurrent resolution has been adopted directing the public printer to print the same. The manuscript is now in the office of the public printer and will be put to press at once under the supervision of the editorial committee, of which Prof. John Guiteras, of Philadelphia, is chairman.

---

The Medical Society of the Women's Medical College of Baltimore has commenced the publication of a bulletin, in which it is proposed to publish the papers, cases and proceedings that come before the society. The first number was issued February 15, 1894, as a four-page double-column quarto sheet. The officers of the society are: President, Fannie E. Hoopes, M. D., D. D. S.; Vice-President, Ida Pollack, M. D.; Recording Secretary, Sue Radcliffe; Corresponding Secretary, Ella J. Reed; Treasurer, Louise Eaton.

---

Messrs. William R. Warner & Company, of Philadelphia, received the award of a silver medal at the late International Medical Congress at Rome.

---

Notice to Contributors.—We are glad to receive contributions from every one who knows anything of interest to the profession. Articles designed for publication in the Journal should be handed in before the first day of the month. The Editors are not responsible for the views or opinions of contributors. All communications should be addressed to the Managing Editor, 284 Franklin St., Buffalo, N. Y.

